# Predicting adolescent depressive symptoms using teacher-reported textual descriptions of abnormal behaviors: a study based on machine learning

**DOI:** 10.3389/frai.2025.1732682

**Published:** 2026-01-08

**Authors:** Nigela Wumaierjiang, Guoli Yan, Lidan Yuan, Jianan Song, Xiaofei Hou, Minghui Li, Ling Sun, Jiansong Zhou, Huifang Yin, Guangming Xu

**Affiliations:** 1 Mental Health Center of Tianjin University, Tianjin Anding Hospital, Tianjin, China; 2 Department of Psychiatry, National Clinical Research Center for Mental Disorders, and National Center for Mental Disorders, The Second Xiangya Hospital of Central South University, Hunan, China

**Keywords:** adolescent depression, machine learning, prediction model, random forest, teacher report

## Abstract

**Objective:**

This study aimed to develop and compare machine learning (ML) models for predicting depressive symptoms in adolescents, based on teacher-reported textual descriptions of student behaviors.

**Methods:**

Participants were 441 adolescents from Tianjin, China. Their teachers provided written reports on behavioral or emotional concerns, while the students completed the Patient Health Questionnaire-9 (PHQ-9). Text data from reports were processed using Term Frequency-Inverse Document Frequency (TF-IDF). Four ML models—Random Forest (RF), Support Vector Machine (SVM), eXtreme Gradient Boosting (XGBoost), and Least Absolute Shrinkage and Selection Operator (LASSO)—were trained and evaluated using a 80/20 data split and 5-fold cross-validation.

**Results:**

PHQ-9 screening identified 71.7% (*n* = 316) of adolescents with clinically significant depressive symptoms (score ≥10). The Random Forest (RF) model demonstrated superior performance, achieving a recall of 0.97, accuracy of 0.91, precision of 0.92, and F1-score of 0.92. SVM and XGBoost also showed good performance, while LASSO was the weakest. The analysis demonstrated that teacher reports could identify depressive symptoms with up to 97% recall.

**Conclusion:**

Machine learning, particularly Random Forest, can effectively predict adolescent depressive symptoms from teacher-reported text. This approach offers a practical and efficient tool for early identification in school settings, facilitating timely intervention.

## 1 Introduction

Depressive disorder has significant negative impacts on adolescents. According to a 2024 report by the World Health Organization (WHO), the current prevalence of depression is 1.4% among adolescents aged 10–14 and 3.5% among those aged 15–19 globally (WHO, 2024, October 10th). Data from China’s mental health surveys further reveal that 14.8% of adolescents exhibit depressive symptoms, with the prevalence of depressive disorders meeting DSM-5/ICD-11 diagnostic criteria ranging from 2.29 to 7.4% (Chinese Academy of Sciences, 2022). Beyond its immediate detrimental effects on adolescent social functioning and academic development, depressive mood is strongly associated with an elevated risk of developing major depressive disorder and profoundly impacts mental health and psycho-social outcomes in young adulthood (Psychogiou et al., 2024). Furthermore, adolescents with untreated depressive disorder face substantial suicide risk and contribute significantly to disability burden. Depressive disorders, together with anxiety disorders, account for 22.4% of the total disability burden attributable to mental disorders in this population in China (Dong et al., 2025).

However, the identification of depressive mood in adolescents currently faces manifold difficulties. The 2021–2022 Mental Health Literacy Survey Report indicates that adolescents’ recognition rate of depressive disorders is only 12.3%, significantly lower than other age groups and without improvement across educational stages. Individuals with high mental health literacy demonstrate better identification of psychological disorders. Nevertheless, most adolescents lack knowledge on appropriately addressing mental health issues when encountered. Deficiencies in mental health literacy and experience among parents, teachers, and others also impede adolescents’ recognition of psychological disorders. This leads to delayed intervention, missed optimal windows for early treatment, and potential psychological crises (Phoa et al., 2023). Furthermore, stigma constitutes one of the most significant barriers to recognizing adolescent depressive disorders. Studies show that 80% of respondents strongly believe the impact of stigma and discrimination may be more severe than the mental illness itself (Thornicroft et al., 2022). Another study highlights that stigma associated with depressive disorders negatively correlates with help-seeking intentions among adolescents; perceived stigma reduces their willingness to seek psychological support (Barney et al., 2006). Simultaneously, parental stigma highly correlates with negative reactions toward adolescent depressive symptoms. Higher parental stigma intensifies negative responses to their children’s depressive moods, resulting in concealment of symptoms and treatment delays (Johnco and Rapee, 2018). These global patterns manifest with distinctive characteristics in the Chinese context, where stigma is deeply embedded in collectivist values and family-oriented social structures. The Confucian emphasis on “harmony” and “face” transforms depression from an individual health issue into a matter of family reputation, creating what scholars term “affiliate stigma” (Yan et al., 2025; Yang et al., 2020). This cultural framework produces several unique manifestations: Chinese adolescents demonstrate greater sensitivity to public than personal stigma(Wei et al., 2023); they tend to express psychological distress through somatic symptoms like insomnia and fatigue, consistent with traditional views of emotional problems as “qi and blood imbalance” (Yan et al., 2025); and family support systems often paradoxically reinforce stigma through “controlling care” aimed at protecting family reputation (Yang et al., 2020).

Although instruments like the PHQ-9 remain indispensable, their large-scale deployment in ordinary schools collides with stubborn realities. Depressed adolescents often fear stigma and either opt out or mask their distress, introducing systematic response bias; coordinating mass testing drains instructional minutes and scarce specialist hours, making repeat waves hard to justify; and the snapshot captures only how a student feels on one particular morning, missing the day-to-day behavioral drift that signals emerging disorder.

Teacher narratives, harvested from the flow of lessons, flip these constraints into advantages. First, ecological validity: every comment is grounded in the authentic contexts of group work, hallway transitions, and homework feedback—functional change observed where it naturally occurs. Second, early-alert leverage: seasoned teachers notice micro-shifts—withdrawal from peers, slowed cognitive tempo, irritability spikes—weeks before those changes would meet a cut-off on a self-report form, widening the window for low-intensity intervention. Third, built-in sustainability: the act of noticing and jotting is already part of pedagogical life; no bells are suspended, no extra staff are hired, and the incremental workload is negligible.

Consequently, the teacher-report stream is not positioned against the PHQ-9 but upstream from it, forming the first step of a stepped-care funnel: continuous observation surfaces “worth-watching” students, algorithmic triage converts those impressions into a ranked risk list, and standardized scales plus clinical interview follow only for the narrowed cohort. Observation begets attention; attention begets precision assessment—an efficient, stigma-sparing cascade that fits the rhythms of a real school.

The assessment methods for adolescent depressive disorders primarily include self-reporting, informant reports, and clinical interviews. However, these approaches exhibit several inherent limitations:

1. Some instruments emphasize somatic symptoms (e.g., fatigue, sleep disturbances) while neglecting emotional fluctuations(e.g., irritability, hedonism). Others exclude somatic symptoms entirely, potentially overlooking cases where depression manifests predominantly through physical complaints (e.g., headaches, gastrointestinal issues), which is common in adolescents.
2. Lengthy scales (e.g., Reynolds Adolescent Depression Scale [RADS], Children’s Depression Inventory [CDI]) may induce respondent fatigue, reducing response accuracy. Brief tools (e.g., PHQ-2, Kutcher Adolescent Depression Scale-6 [KADS-6]) save time but risk omitting critical symptoms like suicidal ideation or functional impairment.
3. All tools rely on single-time-point responses, failing to capture symptom evolution (e.g., mood reactivity to stressors) or longitudinal patterns. They lack mechanisms to incorporate real-time behavioral observations or contextual triggers (e.g., academic pressure, social conflicts).
4. Clinical interviews require trained professionals to differentiate depression from similar conditions. This limits scalability in resource-constrained settings, where staff may lack specialized diagnostic skills.

Schools serve as the primary socialization venue for adolescents. Teachers hold a unique advantage in mental health monitoring due to their high-frequency, multi-dimensional observation perspective. During the teaching process, teachers need to engage in deliberate classroom observation. Through this process, they are able to observe the expressions, behaviors, and verbal communications of individual students, thereby summarizing their personal characteristics, personality traits, and behavioral patterns (Anran Li and Jinju, 2018). However, existing teacher assessment tools exhibit two significant deficiencies: an over-reliance on structured scales, which impedes the effective extraction of semantic features from free-text reports; and a lack of dynamic analytical mechanisms, making it difficult to promptly identify evolving emotional trajectories.

Machine learning technologies offer a promising pathway to overcome these limitations. Algorithms such as Random Forest(RF) and Support Vector Machine(SVM) (Tabares Tabares et al., 2024) have been successfully applied to predict anxiety and depression in adolescents; their nonlinear modeling capabilities can effectively parse multi-source heterogeneous data. This study innovatively introduces natural language processing techniques to analyze teacher textual reports: semantic mining is employed to extract potential predictors of mood, behavior, and social functioning, thereby constructing a predictive model for depressive mood. Compared to traditional methods, this paradigm offers three distinct advantages: it reduces dependence on standardized scales, enabling non-intrusive assessment; enhances screening efficiency through automated analysis; and leverages the continuous nature of teacher observations to facilitate dynamic tracking.

This research explores an auxiliary tool based on teacher observations, aimed at assisting educators in conducting preliminary assessments and prioritization of depression risks among students of concern. The anticipated outcome is specifically adapted for educational settings to address limitations in existing assessment systems, such as the lack of scalable and objective means to triage students needing further support. Furthermore, this research will provide critical technical support for constructing a three-level, intervention-oriented early warning mechanism: “Teachers’ observation →Intelligent Analysis→ Professional Intervention.” This framework is designed to facilitate the early identification and timely intervention of adolescent mental health concerns.

## 2 Methods

### 2.1 Participants

The study population was derived from the Tianjin Mental Health Promotion Program for Children and Adolescents in Tianjin, China, aimed at investigating the mental health status of middle school students. Data were collected via an online questionnaire survey administered from September 2024 to January 2025 using a convenience sampling approach. The survey was distributed through the WJX platform.^1^ Prior to the survey, informed consent forms were provided by homeroom teachers to both students and their parents. Upon obtaining consent from both parties, homeroom teachers completed a self-designed teacher questionnaire to identify students with reported behavioral or emotional concerns. These selected students then completed a basic information form and the Patient Health Questionnaire-9 (PHQ-9).

A total of 606 valid questionnaires were collected. After further screening, the final sample comprised 441 teacher-completed questionnaires and their corresponding 441 student self-administered questionnaires. All 441 teacher questionnaires were completed by a total of 178 teachers. Among the students, 183 (41.5%) were male and 258 (58.5%) were female. The age range of the students was 12 to 18 years, with a mean age of 15.3 years.

#### 2.1.1 Sample size calculation

To ensure the reliability of research findings and the validity of statistical analyses, the sample size for this study was calculated based on the anticipated performance of the predictive model. Given that the study aims to classify students’ depressive symptoms using a machine learning model, the sample size estimation was conducted using an accuracy-based approach.

Assuming an expected model accuracy of 80%, a maximum permissible error margin of 5%, and a 95% confidence level (corresponding to a Z-value of 1.96), and the minimum required sample size was calculated using the following formula:

$$n=\frac{Z^{2}.p.(1-p)}{E^{2}}$$

Where:

*n* = required sample size.

Z = Z-value for the confidence level (1.96).

p = expected accuracy (0.8).

E = acceptable error margin (0.05).

Substituting these parameters, the minimum required sample size was determined to be 246. The actual sample size collected in this study was 441, which substantially exceeds the calculated minimum, ensuring sufficient statistical power to meet the research objectives and analytical requirements.

### 2.2 Research instruments

#### 2.2.1 Teacher questionnaire: self-developed teacher report form

The teacher-reported questionnaire collected the following information:

Teacher details: Name and contact information.

Student demographics: Name, grade, class, gender, and age.

Semi-open-ended questions: assessing student behavioral and psychological concerns:

1. *“Please describe any observed abnormalities in the student’s emotional state, interpersonal relationships, academic performance, family situation, or physical health*.*”*
2. Duration of the reported issues
3. Severity of the issues’ impact

Teachers were invited to participate in the study by submitting reports on students who they perceived as experiencing significant difficulties. Specifically, they were instructed to select up to five students who currently concerned them the most based on their professional judgment of the students’ overall wellbeing, with ‘difficulties’ operationalized as observable challenges in the emotional, interpersonal, academic, familial, or physical health domains. Each report then required the teacher to respond to the semi-open-ended questions detailed below. This data collection strategy yielded a rich corpus of textual data focused on teacher-identified at-risk students, which was used for subsequent feature extraction and model training.

#### 2.2.2 Student questionnaire

##### 2.2.2.1 Basic demographic information form

Students self-reported their demographic details, including: Name, Gender, Date of birth, Grade, Class designation.

##### 2.2.2.2 Patient health questionnaire-9 (PHQ-9)

PHQ-9 is a 9-item self-report depression screening tool (Kroenke et al., 2001) with each item corresponding to one of the nine diagnostic criteria for major depressive disorder (MDD) in DSM-IV. Responses are scored on a 4-point Likert scale (0–3), with the total score ranging from 0 to 27. In the Chinese version of the PHQ-9, the Cronbach’s *α* coefficient was 0.85 in an adolescent population, indicating good internal consistency (Wang et al., 2014). The cut-off score of 10 was chosen for this study based on findings that it maximizes both sensitivity and specificity (Negeri et al., 2021). For the purposes of this study, scores of 0–9 were classified as “no depressive symptoms” (negative case, coded as 0), while scores of 10–27 were classified as “depressive symptoms present” (positive case, coded as 1).

### 2.3 Data analysis

#### 2.3.1 Data preprocessing

The preprocessing phase involved fundamental data cleaning and feature extraction. Data cleaning included the removal of punctuation, special characters, and stop words. Given the significant class imbalance in our dataset (316 negative cases vs. 125 positive cases), we applied oversampling techniques to the minority class to prevent model bias and improve the detection of at-risk individuals. Feature extraction was performed using the Term Frequency-Inverse Document Frequency (TF-IDF) method to transform textual data into numerical representations suitable for machine learning models.

#### 2.3.2 Model selection

Four machine learning models were selected for this study: 1. Random forest (RF), 2. Support vector machine (SVM), 3. eXtreme gradient boosting (XGBoost), and 4. Least absolute shrinkage and selection operator (LASSO). The core characteristics and optimization strategies of these models are summarized in Table 1.

**Table 1:** Core characteristics and optimization strategies of models used in this study.

Model name	Core characteristics	Optimization strategies
Random forest (RF)	An ensemble learning method that improves prediction accuracy and stability by constructing multiple decision trees and aggregating their outputs. Makes minimal assumptions about data distribution, suitable for diverse data types, and capable of handling high-dimensional features.	Tuning hyper parameters including: number of trees, maximum depth, and minimum samples required for node splitting.
Support vector machine (SVM)	A supervised learning model that identifies an optimal hyper plane to maximize the margin between different classes. Employs kernel tricks to map data into higher-dimensional spaces for nonlinear problem solving. Particularly effective when the number of features exceeds sample size, demonstrating strong generalization capability.	Selecting appropriate kernel functions (e.g., linear or RBF kernels) and tuning the regularization parameter (C value).
XGBoost (eXtreme Gradient Boosting)	A gradient boosting-based ensemble method that sequentially builds weak learners to optimize the loss function. Features fast training speed and robust handling of missing values/outliers.	Optimizing hyper parameters: number of trees, tree depth, learning rate, and regularization terms.
LASSO (Least Absolute Shrinkage and Selection Operator)	A linear regression model incorporating L1 regularization for feature selection, enabling sparse representation by shrinking some coefficients to zero. Provides model simplicity and high interpretability.	Adjusting regularization strength (α value) to balance bias-variance trade off.

Rationale for model selection:

- High-dimensional text data: RF, SVM, and XGBoost are particularly effective in handling high-dimensional feature spaces, which are common in textual analysis.
- Nonlinear relationships in mental health prediction: SVM leverages kernel tricks to capture complex nonlinear patterns, while XGBoost employs gradient boosting to model intricate relationships in the data. LASSO was included for its feature selection capabilities, which help mitigate over-fitting in high-dimensional scenarios.

#### 2.3.3 Model training and validation

Data splitting

The dataset was partitioned into an 80% training set and a 20% test set. To ensure model stability and generalization capability, 5-fold cross-validation was additionally employed for robust performance evaluation.

#### 2.3.4 Model evaluation

Appropriate evaluation metrics are essential for assessing model generalizability. In binary classification, predictions can be categorized into:

True Positives (TP), True Negatives (TN), False Positives (FP), False Negatives (FN).

The following metrics were adopted:

Recall (Sensitivity/True Positive Rate).

Measures the model’s ability to correctly identify positive cases (depressive symptoms in this study):

$$\text{Recall}=\frac{\mathrm{TP}}{\mathrm{TP}+\mathrm{FN}}$$

Accuracy.

Quantifies overall prediction correctness:

$$\text{Accuracy}=\frac{\mathrm{TP}+\mathrm{TN}}{\mathrm{TP}+\mathrm{FP}+\mathrm{TN}+\mathrm{FN}}$$

Precision.

Indicates the proportion of correctly predicted positives among all predicted positives:

$$\text{Precision}=\frac{\mathrm{TP}}{\mathrm{TP}+\mathrm{TN}}$$

F1 Score.

Harmonic mean of precision and recall, particularly suitable for imbalanced datasets:

$$F1\;\text{Score}=2\times \frac{\text{Precison}\times \text{Recall}}{\text{Precison}+\text{Recall}}$$

The Receiver Operating Characteristic curve (ROC curve) & The Area Under the Curve (AUC).

ROC curve plots True Positive Rate (TPR) against False Positive Rate (FPR) across classification thresholds. AUC provides a scalar measure of model discrimination, where higher values indicate superior performance.

#### 2.3.5 Feature importance evaluation

To enhance the interpretability of the machine learning models and identify key textual features associated with depressive symptoms, we employed SHapley Additive exPlanations (SHAP) analysis. SHAP is a game-theoretic approach that assigns each feature an importance value for individual predictions, providing a unified measure of feature impact across models. This method was applied to all four models: RF, SVM, XGBoost, LASSO.

Specifically, for each model, we calculated the mean absolute SHAP values for all features derived from the TF-IDF vectorization (e.g., top 20 words from teacher reports). Features were ranked by their mean SHAP values to determine global importance. Additionally, we analyzed SHAP summary plots to visualize the direction of feature impacts (e.g., whether high frequency of a word like “unhappy” increased or decreased depression risk). This approach allowed us to compare feature importance consistently across linear and nonlinear models, addressing potential biases in single-model interpretations. The analysis was implemented using the SHAP library in Python, with results reported in the Results section.

## 3 Results

### 3.1 Teachers’ reports

**Figure 1:**
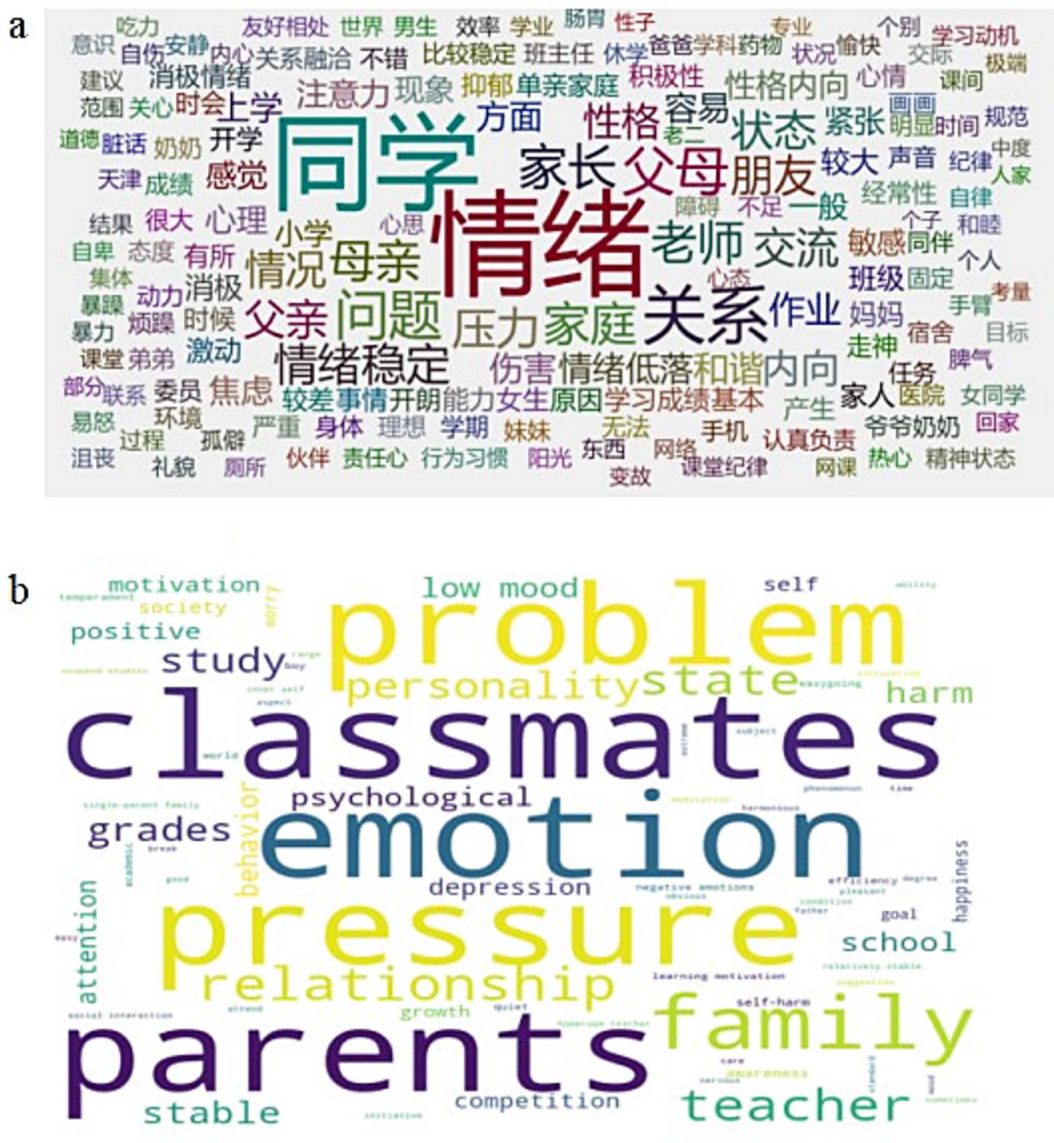
Word frequency statistics of teachers’ report texts. The **(a)** presents the word cloud generated directly from the original Chinese teacher reports, reflecting the actual frequency of key terms in the dataset. The **(b)** is provided for the convenience of international readers; it is not a word cloud generated from translated text but a visual reference where the Chinese terms from the left cloud are replaced with their English translations, preserving the original frequency relationships. This approach ensures the integrity of the Chinese linguistic analysis while enhancing accessibility.

A total of 441 teachers’ reports were completed by 178 teachers, with a minimum of 1 report and a maximum of 5 reports per teacher. For the text reports we selected the top 1,000 words with the highest word frequency for word frequency statistics, as shown below (Figure 1).

The word frequency analysis revealed that the high-frequency terms in teachers’ reports were primarily concentrated in three core dimensions: emotional state (e.g., “low mood,” “unhappy”), social behavior (e.g., “unwilling to communicate,” “alone”), and academic performance (e.g., “inattention,” “declining grades”). This distribution pattern indicates that teachers’ observational perspectives naturally cover the common external manifestation domains of adolescent depressive symptoms, providing preliminary validity evidence for using their textual descriptions in depression prediction. These high-frequency words formed the basis for subsequent feature extraction in the machine learning models.

### 3.2 Adolescent depression identification rate

In this study, the results of the PHQ-9 completed by students were used as an indicator of the presence of depression symptoms in adolescents, who were categorized as having or not having depression symptoms based on the questionnaire scores. As detailed in Table 2, the PHQ-9 screening indicated that a majority of the adolescent participants (*n* = 316, 71.7%) exhibited clinically significant depressive symptoms (scores≥10, categorized as 1).

**Table 2:** Distribution of depression among adolescents.

Variables	Category	Without	With	Total
Gender	Male	60(13.6%)	123(27.8%)	183
	Female	65(14.7%)	193(43.8%)	258
Grades	7	52(11.8%)	109(24.7%)	161
	8	25(5.7%)	52(11.8%)	77
	9	12(2.7%)	66(14.9%)	78
	10	22(5.0%)	54(12.2)	76
	11	12(2.7%)	16(3.6%)	28
	12	2(0.5%)	19(4.3%)	21
Ages	12	31(6.3%)	60(13.7%)	91
	13	28(6.3%)	75(17.0%)	103
	14	19(4.3%)	56(12.7%)	75
	15	17(3.9%)	37(8.4%)	54
	17	10(2.3%)	27(6.1%)	37
	18	1(0.2%)	11(2.5%)	12

Note: Percentages here are cases as percentages of the total.

For the purpose of this study, correctly identifying data with depression symptoms as having depressive symptoms (categorized as 1) is considered to be the models’ ability to correctly identify adolescent depression. Therefore when reporting recall, this study will only report recalls for classification 1.

**Figure 2:**
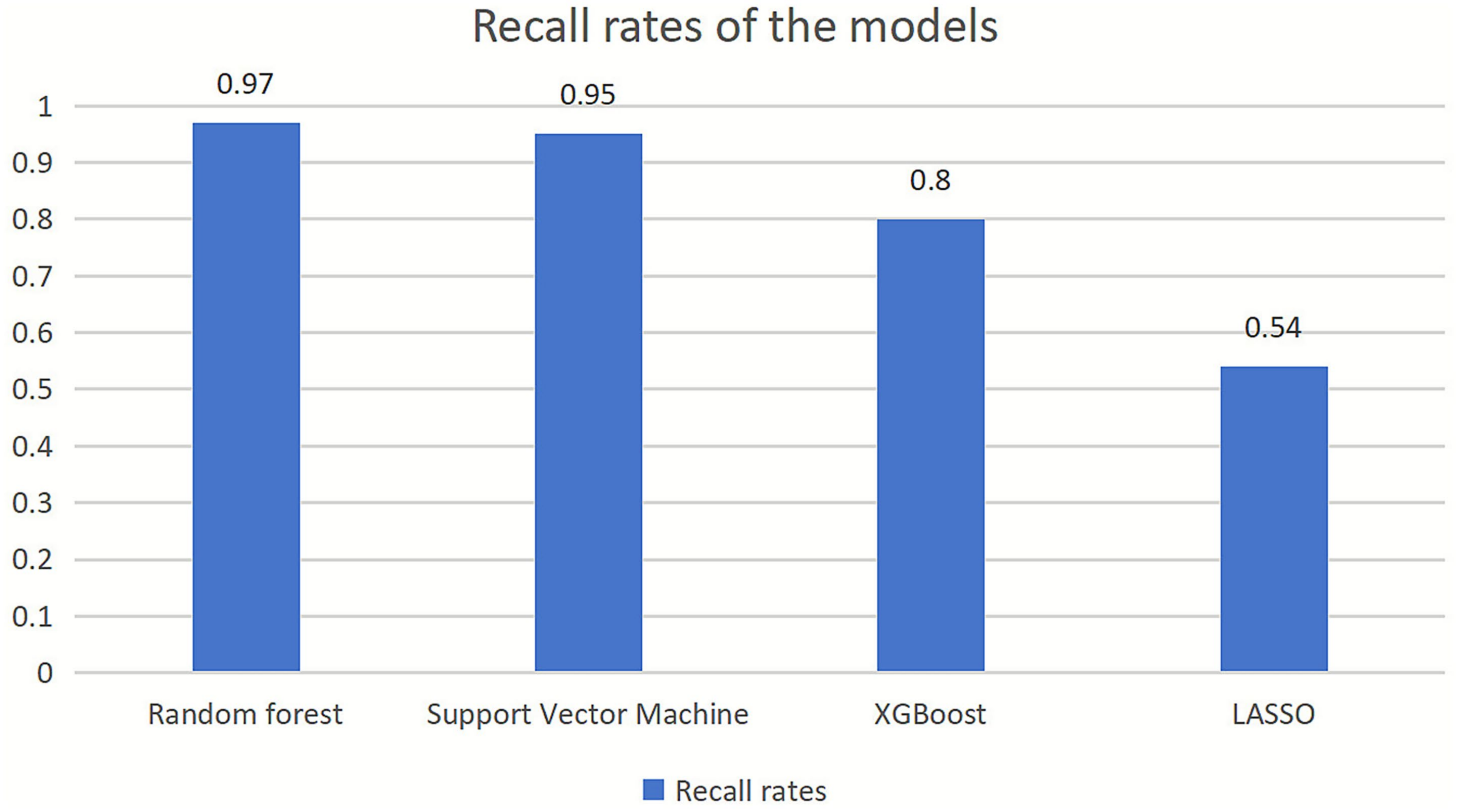
The recall rates (recall for Class 1) of the models.

The recall rates (recall for Class 1) of the four models in this study are illustrated in Figure 2.

The detection rate of teacher-reported texts for adolescent depressive symptoms reached up to 97%. Specifically, among samples with depressive symptoms, 97% of those exhibiting symptoms were correctly identified as “having depressive symptoms.”

### 3.3 Model performance comparison

In addition to recall, the accuracy, precision, and F1-score of the four models were evaluated. Due to the imbalanced class distribution in the dataset, weighted average metrics were adopted for assessment (Table 3).

**Table 3:** Evaluation metrics of the models.

Model	Accuracy	Precision	F1-score
Random Forest	0.91	0.92	0.92
Support Vector Machine	0.83	0.85	0.83
XGBoost	0.84	0.85	0.84
Lasso	0.66	0.67	0.66

As indicated by the data in the table, the RF model achieved the best performance across all metrics, followed by XGBoost and SVM, while the Lasso model demonstrated comparatively inferior results.

The RF model attained an accuracy of 0.91, precision of 0.92, recall (for Class 1) of 0.97, and an F1-score of 0.92. These results highlight its high reliability and effectiveness in predicting adolescent depressive symptoms. The notably high recall rate is particularly critical, as it ensures the identification of the majority of adolescents with depressive symptoms, which holds significant implications for early intervention and treatment.

The SVM model achieved an accuracy of 0.83, precision of 0.85, and F1-score of 0.83. Although its recall rate approached that of RF, its overall performance was slightly weaker. This may stem from SVM’s limitations in handling imbalanced class data, particularly in high-dimensional text data, where kernel function selection and parameter tuning substantially influence performance.

The XGBoost model yielded an accuracy of 0.84, precision of 0.85, and F1-score of 0.84. While XGBoost excels in capturing nonlinear relationships and processing high-dimensional data, its recall rate was marginally lower than those of RF and SVM. This suggests a potential risk of missing true positive cases when identifying adolescents with depressive symptoms, despite its overall robust performance.

The Lasso model exhibited the poorest performance, with accuracy, precision, and F1-score all at 0.66. As a linear model, Lasso demonstrates limited capacity to address nonlinear relationships and is highly sensitive to feature correlations. Furthermore, its performance shortcomings may be exacerbated by class imbalance within the dataset.

To provide a more intuitive visualization of model performance, we plotted the confusion matrices and ROC curves for each model. The confusion matrices detail the prediction outcomes across classes, including True Positives (TP), False Positives(FP), True Negatives(TN), and False Negatives(FN). These matrices enable clearer identification of misclassification patterns (Table 4).

**Table 4:** The confusion matrices of the random forest model.

Actual/Predicted	Predicted not depressed	Predicted depressed
Actual not depressed	83	42
Actual depressed	10	306

The confusion matrix for the RF model revealed a high TP-to-FN ratio in identifying adolescents with depressive symptoms, demonstrating its exceptional performance in minimizing missed detection.

The confusion matrices for SVM and XGBoost models also exhibited relatively high TP proportions. However, their FN ratios slightly exceeded those of RF, suggesting potential risks of under detection in identifying symptomatic adolescents.

In contrast, the Lasso model displayed a notably higher FN proportion, indicating substantial under detection of depressive symptoms, which may explain its lower recall rate.

The ROC curves illustrate classification performance by plotting the TPR against the FPR across varying thresholds. A higher AUC value indicates better classification performance. RF achieved the highest AUC value, confirming its superior discriminative ability in distinguishing between adolescents with and without depressive symptoms. XGBoost and SVM also attained high AUC values, albeit marginally lower than RF. The Lasso model yielded the lowest AUC value, reflecting its limited classification efficacy.

In summary, the RF model demonstrated optimal performance in identifying adolescent depressive symptoms, with its high recall rate and F1-score establishing it as the most robust model in this study. Nevertheless, limitations persist across models: Lasso’s inadequacy in handling nonlinear relationships, and the slightly inferior performance of XGBoost and SVM in class-imbalanced data compared to RF.

### 3.4 Feature importance analysis

To elucidate the predictive basis of the models and identify the key behavioral and psychological cues teachers used, we conducted a feature importance analysis using SHAP. The top predictive features (words) for each model, ranked by their mean absolute SHAP values, are summarized in Table 5.

**Table 5:** Top 5 most important features and their mean SHAP values for the four models.

Model (SHAP value)	Rank 1	Rank 2	Rank 3	Rank 4	Rank 5
Random forest	Hyperactive (0.0125)	Dislike (0.0125)	Recent (0.0112)	Conflict (0.0100)	Self (0.0095)
XGBoost	Relationship (0.0336)	Emotion (0.0304)	Dislike (0.0242)	Student (0.0235)	Classmate (0.0233)
SVM	Dislike (0.0017)	Active (0.0013)	Attend Class (0.0013)	Relationship (0.0009)	Interpersonal (0.0008)
LASSO	Pressure (0.0078)	Recent (0.0060)	Get Along (0.0055)	Family (0.0050)	Child (0.0045)

As shown in Table 5, the optimal RF model highlighted terms related to externalizing behaviors and recent changes, such as “hyperactive,” “dislike,” and “recent.” The XGBoost model assigned the highest importance to words describing interpersonal dynamics, including “relationship,” “emotion,” and “classmate.” The SVM model’s top features were a mix of behavioral (“dislike,” “active”) and academic/interpersonal terms (“attend class,” “relationship”). In contrast, the linear LASSO model emphasized more abstract or contextual concepts like “pressure,” “recent,” and “family.”

Notably, the term “dislike” consistently appeared among the top features across three models (RF, XGB, SVM), suggesting its robust association with depressive symptoms in teachers’ observational reports.

## 4 Discussion

The teacher-report analytic pipeline developed in this study is designed to complement, not compete with, the PHQ-9. The Random-Forest model’s 97% recall shows that educators’ routine observations reliably capture depression-related behaviors, offering real-time, context-embedded detection that the PHQ-9 cannot provide. In everyday school practice, an optimal screening architecture should therefore be tiered: teachers’ daily logs serve as the first gate (the present method) to flag students who warrant attention; standardized instruments such as the PHQ-9 act as a second gate to refine the risk estimate for these pre-selected youths; and clinical interviews constitute the final gate for formal diagnosis. This division of labor maximizes both efficiency and accuracy.

The feature importance analysis deciphers the key behavioral cues from teachers’ reports that drive the models’ predictions. The prominence of terms like “dislike” and “hyperactive” indicates that teachers are effectively capturing non-stereotypical manifestations of adolescent depression, such as irritability and anhedonia, thereby affirming the ecological validity of their observations.

Furthermore, the high importance of interpersonal words like “relationship” and “conflict” underscores the central role of social functioning, while the LASSO model’s focus on “pressure” and “family” highlights the perceived impact of academic and familial stressors, which is particularly salient in the Chinese context.

The consistent significance of “dislike” across multiple models is especially notable, suggesting that a student’s expressed aversion is a highly reliable indicator. This key semantic cue could be prioritized in future teacher training to sharpen early detection efforts.

The necessity of this approach is grounded in the operational realities of schools. Universal PHQ-9 administration is often impractical: it disrupts class time, risks stigmatizing non-participants, and demands scarce mental-health staff for scoring and interpretation. Teacher-report analytics, by contrast, piggybacks on work already embedded in the teaching routine—educators simply record their ongoing observations, and the system automatically surfaces high-risk students. In this way professional resources are targeted only where they are most needed, without adding extra burden to staff or students.

Previous text-based depression detection models commonly utilize electronic health records (Dai et al., 2021), social media texts (Kim et al., 2020), or speech transcriptions. Widely employed traditional machine learning models include Logistic Regression (LR), Decision Tree (DT), Naive Bayes (NB), SVM, and RF. Common evaluation metrics encompass accuracy, AUC value, and F1-score, with accuracy and F1-score typically exceeding 80%, and AUC values reaching 0.9 or higher.

This study’s 97% detection rate demonstrates that the rich behavioral cues embedded in teachers’ narrative reports can effectively reveal depressive symptoms. Crucially, this naturalistic-observation approach sidesteps the social-desirability bias inherent in self-report scales—a particular advantage for adolescents who tend to deny or mask their distress. Among the 441 teacher submissions, educators documented multidimensional behavioral shifts in peer interaction, academic engagement, and emotional expression. These everyday functional changes constitute the real-world manifestation of depression, complementing the subjective experiences captured by the PHQ-9 and providing a fuller, ecologically valid picture for early identification and support.

## 5 Limitations

Several limitations of this study should be acknowledged, as they define the boundaries of our current findings and point to valuable future directions.

First, the sampling strategy inherently influences the generalizability of the model. Participants were selected by teachers based on their perceptions of students needing attention, resulting in a sample with a high prevalence of depressive symptoms. While this limits the model’s applicability for universal screening across an entire student population, it accurately reflects the intended use case of this tool: serving as an aid for teachers to triaging and assessing risk within their already-identified group of concerned students. Thus, the results validate the model’s efficacy within this specific, pragmatically defined context.

Second, reliance on a single data source (teacher reports) presents an incomplete picture. Teachers cannot observe students’ behaviors at home or in the community, and their reports may contain subjective biases. Future research should aim to integrate multi-source data, such as parent reports, student self-reports (where feasible), and anonymized school behavioral records (e.g., attendance, participation), to construct a more comprehensive and robust assessment profile.

Third, although we performed feature importance analysis, further steps could be taken to enhance model interpretability. Techniques like SHAP (SHapley Additive exPlanations) could be employed in the future to elucidate complex feature interactions and provide individual-level explanations, making the model’s outputs more actionable for school personnel.

Finally, the current model is static. Adolescent mental health is dynamic, and symptoms evolve over time. The logical next step is to develop longitudinal models using time-series data. This would enable the tracking of behavioral trajectories, moving from cross-sectional risk identification to truly dynamic forecasting and early warning, allowing for even more timely intervention.

## 6 Conclusion

This study demonstrates that machine learning models, particularly Random Forest, can effectively identify adolescents at risk of depressive symptoms by analyzing teachers’ naturalistic textual descriptions of student behaviors. The high recall rate achieved underscores that the rich behavioral cues embedded in teachers’ routine observations constitute a highly sensitive indicator for preliminary risk assessment.

The primary significance of this research lies in its pragmatic contribution to school mental health practices. It provides a feasible and sustainable methodology that transforms subjective teacher observations into an objective, data-driven auxiliary tool. This approach is not intended to replace standardized scales but to optimize the screening pipeline by enabling efficient triage. It allows school professionals to focus their expertise and resources on students who need them most, thereby promoting early identification within a naturalistic school setting.

Looking forward, the integration of teacher-reported data with advanced analytics represents a promising step toward building dynamic, multi-modal early warning systems. Future efforts should be directed toward validating this approach in more diverse populations, incorporating longitudinal data to capture symptom evolution, and ultimately integrating it with other data sources to provide a more comprehensive understanding of student wellbeing. By continuing to bridge the gap between educational practice and mental health expertise, such tools have the potential to significantly impact the prevention and early intervention of adolescent depression.

## Data Availability

The raw data supporting the conclusions of this article will be made available by the authors, without undue reservation.

## Footnotes

1
https://www.wjx.cn/app/survey.aspx
